# Biological Actions of Bile Acids via Cell Surface Receptors

**DOI:** 10.3390/ijms26115004

**Published:** 2025-05-22

**Authors:** Yoshimitsu Kiriyama, Hiroshi Tokumaru, Hisayo Sadamoto, Hiromi Nochi

**Affiliations:** 1 Kagawa School of Pharmaceutical Sciences, Tokushima Bunri University, Takamatsu 760-8542, Japan; tokumaruh@kph.bunri-u.ac.jp (H.T.); sadamotoh@kph.bunri-u.ac.jp (H.S.); nochi@kph.bunri-u.ac.jp (H.N.); 2 Institute of Neuroscience, Tokushima Bunri University, Takamatsu 760-8542, Japan

**Keywords:** bile acid, gut microbiota, gut microbiome, TGR5, GPBAR, S1P2R, muscarinic receptor, MRGPRX4, bitter taste receptor, TAS2R, LIFR

## Abstract

Bile acids (BAs) are synthesized in the liver from cholesterol and are subsequently conjugated with glycine and taurine. In the intestine, bile acids undergo various modifications, such as deconjugation, dehydrogenation, oxidation, and epimerization by the gut microbiota. These bile acids are absorbed in the intestine and transported to the liver as well as the systemic circulation. BAs can activate many types of receptors, including nuclear receptors and cell surface receptors. By activating these receptors, BAs can exert various effects on the metabolic, immune, and nervous systems. Recently, the detailed structure of TGR5, the major plasma membrane receptor for BAs, was elucidated, revealing a putative second BA binding site along with the orthosteric binding site. Furthermore, BAs act as ligands for bitter taste receptors and the Leukemia inhibitory factor receptor. In addition, the Mas-related, G-protein-coupled receptor X4 interacts with receptor activity-modifying proteins. Thus, a variety of cell surface receptors are associated with BAs, and BAs are thought to have very complex activities. This review focuses on recent advances regarding cell surface receptors for bile acids and the biological actions they mediate.

## 1. Introduction

**Figure 1:**
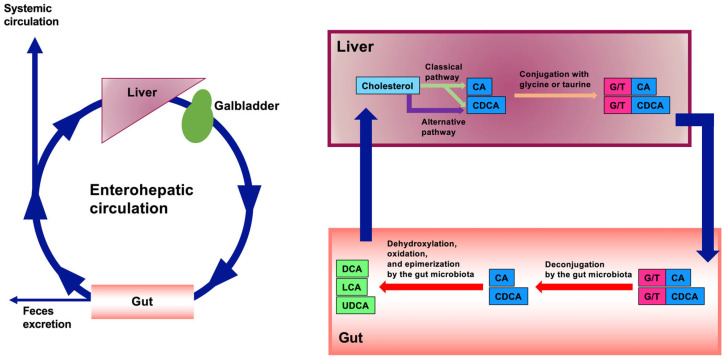
The enterohepatic circulation of bile acids (BAs). BAs are primarily synthesized in the liver from cholesterol through the classical pathway and the alternative pathway. In the classical pathway, the primary BAs, cholic acid (CA) and chenodeoxycholic acid (CDCA), are synthesized. In the alternative pathway, CDCA is synthesized. CA and CDCA are conjugated with glycine or taurine. These BAs are then stored in the gallbladder and secreted into the small intestine upon food intake. Conjugated CA and CDCA are deconjugated by intestinal bacterial bile salt hydrolase. The deconjugated CA and CDCA are then converted by intestinal bacteria to various BAs, such as DCA, LCA, and UDCA. Most BAs absorbed from the intestine are transported to the liver. However, some enter the systemic circulation. BAs that are not absorbed from the intestine are excreted as feces.

Bile acids (BAs) are synthesized from cholesterol in the liver by enzymes such as cytochrome P450 7A1 (CYP7A1) and are called primary BAs. These primary BAs are subsequently conjugated with glycine and taurine [1]. These BAs are then stored in the gallbladder and secreted into the small intestine upon dietary stimulation. Because of their amphipathic steroid structure, BAs form micelles with fat-soluble components, such as cholesterol, lipids, and fat-soluble vitamins, in the intestine to facilitate their absorption. Although most BAs are absorbed in the intestine, BAs that are not absorbed in the intestine undergo various modifications, such as deconjugation, dehydrogenation, oxidation, and epimerization by the gut microbiota during their transport to the colon [2]. These BAs synthesized by the gut microbiota are called secondary BAs [3], which can also be absorbed in the intestine. BAs absorbed in the intestine are transported to the liver through the portal vein. The circulation of BAs between the liver and the intestine is known as enterohepatic circulation. While most reabsorbed BAs return to the liver, a small proportion enter the systemic circulation [3,4] (Figure 1). Moreover, BAs exert a variety of effects on diverse cell types and intracellular organelles, including mitochondria and autophagosomes [5]. Furthermore, BAs can activate many types of receptors, including nuclear receptors, such as the farnesoid X receptor (FXR), and cell surface receptors, such as Takeda G-protein receptor 5 (TGR5) [6]. By activating these receptors, BAs can exert various effects on the metabolic, immune, and nervous systems [1,6,7,8]. Therefore, BAs are now recognized as hormones or signaling molecules. This review focuses on the recent advances in cell surface receptors of BAs and their mediated biological actions.

## 2. Biosynthesis of Various Bile Acids

BAs are primarily synthesized from cholesterol in the liver via two pathways: the classical (or neutral) pathway and the alternative (or acidic) pathway. At least 16 enzymes participate in its biosynthesis in the liver [1,9]. The classical pathway begins with the conversion of cholesterol to 7α-hydroxycholesterol by CYP7A1, which is further converted to 7α-hydroxy-4-cholesten-3-one [10]. The primary BAs, cholic acid (CA) and chenodeoxycholic acid (CDCA), are synthesized from 7α-hydroxy-4-cholesten-3-one by CYP8B1 and CYP27A1, respectively. The alternative pathway involves the conversion of cholesterol to (25R)-26-hydroxycholesterol by CYP27A1, which is further converted to CDCA by CYP7B1 [10]. In mice, CDCA is further converted to α-muricholic acid (MCA) or β-MCA [11]. BAs synthesized in the liver are conjugated to glycine or taurine by BA CoA:amino acid N-acyltransferase (BAAT) [12]. These BAs are then stored in the gallbladder and secreted into the small intestine upon food intake.

BAs conjugated in the liver are deconjugated by intestinal bacterial bile salt hydrolase, which is present in intestinal bacteria, such as the *Lactobacillus* spp. [13]. The deconjugated CDCA and CA are then converted by intestinal bacteria to various BAs. CDCA and CA are converted to 3-oxo-Δ4-LCA and 3-oxo-Δ4-DCA, respectively. 3-oxo-Δ4-LCA and 3-oxo-Δ4-DCA are then converted to 3-oxoLCA and 3-oxoDCA, respectively. Subsequently, 3-oxoLCA is converted to LCA or isoLCA, and 3-oxoDCA is converted to DCA or isoDCA [14,15] (Figure 1).

## 3. Bile Acid Membrane Receptors

GPCRs are cell surface membrane receptors comprising seven transmembrane helices that couple with heterotrimeric G-proteins. GPCRs respond to a wide variety of biological molecules and transmit signals to heterotrimeric G-proteins and arrestins, thereby activating various signaling pathways and cellular functions [16]. Although more than 800 GPCRs are encoded in the genome [16,17], there are more than 100 GPCRs with unknown ligands; these are known as orphan GPCRs [17,18]. TGR5 and Mas-related, G-protein-coupled receptor X4 (MRGPRX4) were once orphan receptors but are now considered to be receptors for BAs.

### 3.1. TGR5

**Figure 2:**
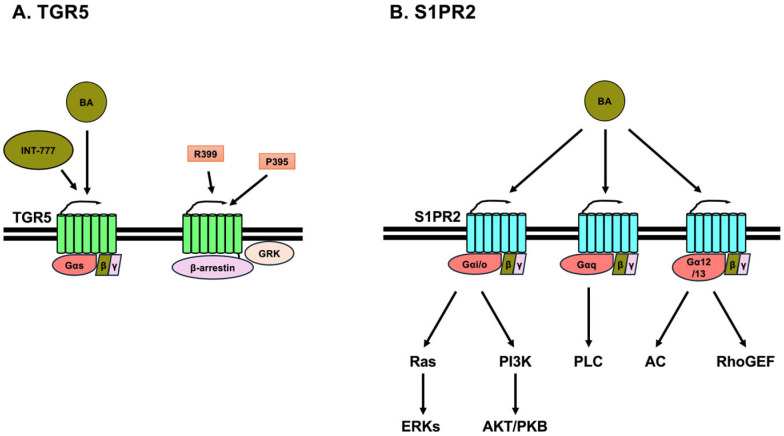
Takeda G-protein receptor 5 (TGR5) and sphingosine-1 phosphate receptor 2 (S1PR2). (**A**) TGR5 primarily couples with Gα_s_, leading to an increase in intracellular cyclic AMP (cAMP) concentration through the activation of adenylate cyclase by Gα_s_. LCA, DCA, CDCA, and CA. Furthermore, the taurine- or glycine-conjugated forms of these bile acids (BAs) activate TGR5. INT-777, a BA derivative that also activates TGR5. The synthetic agonists, P395 and R399, activate TGR5, which in turn activates GPCR-related kinase (GRK), leading to the activation of β-arrestin. (**B**) S1PR2 is associated with Gα_i/o_, Gα_q/11_, and Gα_12/13_. The activation of Gα_i/o_ leads to the activation of phosphoinositide 3-kinase (PI3K) and Ras. PI3K activates AKT/PKB. The activation of Ras leads to the activation of extracellular signal-regulated kinases (ERKs). The activation of Gα_q/11_ leads to the activation of phospholipase C (PLC). The activation of Gα_12/13_ leads to the activation of adenylate cyclase (AC) and Rho guanine nucleotide exchange factors (RhoGEF). TCA, TDCA, GCA, GDCA, and TUDCA are able to activate ERK and AKT/PKB through S1PR2. In contrast, TβMCA inhibits the activation of AKT/PKB and ERK.

TGR5, also known as G-protein-coupled BA receptor 1, membrane-type receptor for BA, or G-protein-coupled receptor 19, has been identified as a GPCR that acts as a cell membrane receptor for BAs [19,20]. The order of potent activation of TGR5 is LCA, DCA, CDCA, and CA. Furthermore, the taurine- or glycine-conjugated forms of these BAs activate TGR5 almost as potently as the unconjugated forms [20]. In humans, TGR5 is highly expressed in the placenta, spleen, and lungs, and moderately expressed in the stomach, small intestine, liver, and adipose tissues [20]. In mice, TGR5 is highly expressed in the digestive tract, including the colon and small intestine, liver, and adipose tissues [21]. Moreover, TGR5 is also expressed in the brain and immune cells [8,20,21,22,23]. TGR5 mainly couples to Gα_s_ [24], and the activation of Gα_s_ leads to an increase in intracellular cyclic AMP (cAMP) levels through the activation of adenylate cyclase by Gα_s_ [25,26] (Figure 2A). In addition, TGR5 has also been reported to be associated with Gα_q_ and Gα_i_ [27,28]. Elevated intracellular cAMP levels lead to the activation of protein kinase A (PKA) and exchange proteins directly activated by cAMP (EPACs) [25,26,29]. Activated PKA and EPACs regulate various signaling pathways, including those of AKT/protein kinase B (PKB), mitogen-activated protein kinases (MAPKs), and transcription factors [25,26]. Activation of TGR5 influences the activities of kinases, such as AKT/PKB [30,31], extracellular signal-regulated kinases (ERKs) [28,32], and transcription factors, such as cAMP response element binding protein (CREB) [32], nuclear factor-kappa B (NF-κB) [33,34], and signal transducer and activator of transcription 3 (STAT3) [35,36]. Furthermore, the activation of TGR5 also leads to the activation of GPCR-related kinase 2 (GRK2) and GRK6, which eventually phosphorylate and activate β-arrestin 1 and 2 (Figure 2A). Activated β-arrestins, in turn, activate SRC kinase to activate antiviral signaling molecules, such as retinoic acid-inducible gene-I, virus-induced signaling adaptor (also known as mitochondrial antiviral signaling), TANK-binding kinase 1, IFN regulatory factor 3, and mediator of IRF3 activation (also known as stimulator of interferon genes) [37]. Cryo-electron microscopy (EM) structural analysis of TGR5 has revealed that TGR5 has an orthosteric binding site and a putative second BA binding site [38]. P395, a synthetic agonist, and INT-777 (6α-ethyl-23(S)-methylchenodeoxycholic acid or S-EMCA), a BA derivative, can bind to the orthosteric binding site. In contrast, BAs bearing a 12-hydroxyl group, such as CA, DCA, GCA, TCA, TDCA, and INT-777, can bind to the putative second BA binding site, resulting in the allosteric coupling between TGR5 and Gα_s_. TGR5 couples to Gα_s_ through intracellular loop 3 (ICL3) of TGR5 [38]. Furthermore, the α5 helix C-terminus of Gα_s_ can interact with TGR5 [24,38]. In contrast, P395 induces β-arrestin activation. The structures of extracellular loop 1 (ECL1), ECL2, and ICL1 of INT-777-bound TGR5 are significantly different from those of P395-bound TGR5 [38]. Thus, these differences may affect the biased activation of Gα_s_ or β-arrestin in TGR5 (Figure 2A). R399, another synthetic agonist, activates TGR5, which in turn activates GRKs, leading to the activation of β-arrestin 1 and yes-associated protein (YAP) transcriptional activity. In contrast, INT-777 activates TGR5, which activates Gα_s_, leading to the inactivation of YAP transcriptional activity. Activation of YAP transcriptional activity is associated with the enhancement of non-small cell lung cancer cell tumorigenesis [39]. Therefore, the biased activation of Gα_s_ by INT-777 or β-arrestin by R399 in TGR5 leads to YAP transcriptional activity. Moreover, the difference in activation of TGR5 may affect the proliferation of cancer cells. Because TGR5 is expressed in many tissues and is associated with a variety of diseases, characterizing and understanding its structure and function will lead to the discovery and design of specific agonists that may be useful for treating these diseases.

### 3.2. Sphingosine-1 Phosphate Receptor 2 (S1PR2)

S1PR2 is a member of the S1PR family, comprising S1PR1, S1PR2, S1PR3, S1PR4, and S1PR5. S1PRs are GPCRs that are expressed in a variety of cells, including those in the brain, lung, heart, liver, kidney, bone, and immune system [40,41]. S1PR1 and S1PR5 are primarily associated with Gα_i/o_, whereas S1PR2 and S1PR3 can associate with Gα_q/11_, Gα_i/o_, and Gα_12/13_. S1PR4 is primarily associated with Gα_q/11_ [40,42] (Figure 2B). Thus, S1PR2 signaling is mediated through three different G-proteins. The activation of Gα_q/11_ leads to the activation of phospholipase C (PLC) to produce diacylglycerol and inositol 1,4,5-trisphosphate (IP_3_), leading to Ca^2+^ release from intracellular stores. The activation of Gα_i/o_ leads to the activation of phosphoinositide 3-kinase (PI3K) and Ras. Activated PI3K subsequently activates AKT/PKB and Rac. AKT/PKB then evokes the activation of I kappa B kinases to activate nuclear factor-kappa B. Rac activates mitogen-activated protein kinases to activate c-Jun N-terminal kinase. However, Ras activates Raf-MAP kinase or ERK kinases (MEKs)-ERKs pathway. The activation of Gα_12/13_ leads to the activation of adenylate cyclase and Rho guanine nucleotide exchange factors (RhoGEF). Adenylate cyclase causes elevated cAMP levels, which activate PKA. RhoGEF activates RhoA, leading to the activation of RhoA/Rho kinase (ROCK) and inhibition of Rac [42] (Figure 2B).

The conjugated BAs, TCA, TDCA, GCA, GDCA, and TUDCA, are able to activate ERK and AKT/PKB through S1PR2 in primary rat hepatocytes and mouse cholangiocytes [43,44] (Figure 2B). TCDCA induces the activation of ERK, leading to the secretion of cortisol from H295R cells (human adrenal gland carcinoma cell line). Furthermore, TCDCA induces steroidogenesis-related genes, such as steroidogenic acute regulatory protein, hydroxy-delta-5-steroid dehydrogenase, 3 beta- and steroid delta-isomerase 2, and cytochrome P450 family 21 subfamily A member 2, through the activation of steroidogenic factor 1 in H295R cells [45,46,47]. TCA promotes the proliferation and migration of JX-2 cells (human hepatic stellate cell line) through S1PR2 and induces α-smooth muscle actin and collagen I in JX-2 cells. TCA induces YAP nuclear localization through p38 MAPK in JX-2 cells [48]. Tauro-βMCA (TβMCA) inhibits the expression of S1pr2 and activation of AKT/PKB and ERK in mouse bone marrow-derived macrophages [49]. Furthermore, TβMCA and glycine-βMCA inhibit IL-1β secretion, gasdermin D (GSDMD) cleavage, and lactate dehydrogenase leakage in mouse bone marrow-derived macrophages by inhibiting S1pr2 [49]. The cleaved N-terminal domain of GSDMD forms membrane pores, which lead to pyroptotic cell death [50]. TCA induces S1pr2 expression and reduces RhoA and ROCK1 levels via S1pr2 in HepG2-NIAS cells (human hepatoma cell line) [51,52]. Furthermore, TCA induces the expression of C-C motif chemokine ligand 2, also known as monocyte chemoattractant protein 1, and activates microglia, resulting in inflammation in primary mouse neurons [53]. DCA induces the expression of *lncRNA57RIK*, a long non-coding RNA, through S1PR2 in human macrophages. Gα_i_, PI3K, and AKT/PKB are associated with *lncRNA57RIK* expression. *LncRNA57RIK* binds to caspase 4 to secrete IL-1β [54]. S1PR2 is expressed in various tissues and interacts with the three G-proteins. Moreover, multiple BAs activate S1PR2, suggesting that BA-mediated S1PR2 activation exhibits a complex effect on various tissues.

### 3.3. Muscarinic Acetylcholine Receptors (mAChRs)

**Figure 3:**
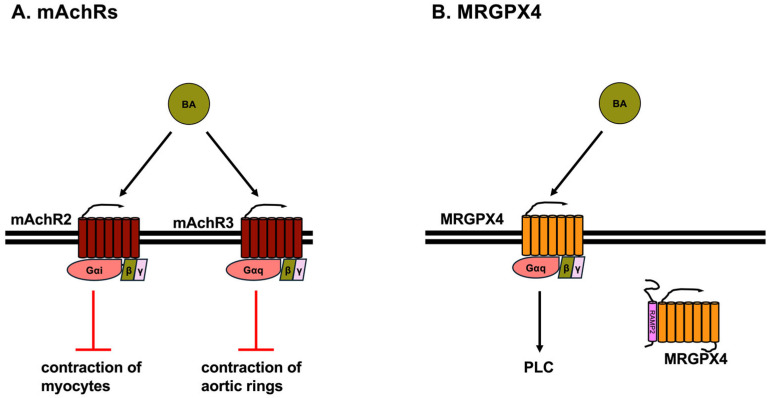
Muscarinic Acetylcholine Receptors (mAChRs) and Mas-related, G-protein-coupled receptor X4 (MRGPRX4) with receptor activity-modifying protein 2 (RAMP2). (**A**) mAChR M2 couples with Gα_i_, whereas mAChR M3 couples with Gα_q_. TCA, TCDCA, TDCA, and GDCA reduce the contraction of myocytes via mAChR M2. TDCA attenuates phenylephrine-induced contraction in aortic rings via mAChR M3. (**B**) MRGPRX4 couples with Gα_q_ and the activation of MRGPRX4 by DCA, TDCA, UDCA, CDCA, TCDA, and CA leads to the activation of phospholipase C (PLC) and Ca^2+^ release. MRGPRX4 can associate with RAMP2, which significantly decreases the cell surface expression of MRGPRX4.

mAChRs are GPCRs and are classified into five subtypes: mAChR M1, mAChR M2, mAChR M3, mAChR M4, and mAChR M5. mAChR M1, mAChR M3, and mAChR M5 are coupled to Gα_q_, whereas mAChR M2 and mAChR M4 are coupled to Gα_i_ [55]. mAChR M2 is expressed in the brain, heart, lung, liver, small intestine, colon, placenta, bladder, and smooth muscle. mAChR M3 is expressed in the brain, exocrine and endocrine glands, heart, lung, stomach, spleen, small intestine, kidney, and smooth muscle [55]. TCA inhibits the contraction of neonatal rat myocytes through Gα_i_ activation, whereas methoctramine, a mAChR M2 antagonist, prevents the inhibition of the contraction of neonatal rat myocytes (Figure 3A). Furthermore, the knockdown of mAChR M2 by siRNA also prevents the TCA-induced inhibition of contraction in neonatal rat myocytes [56]. TCDCA, TDCA, and GDCA reduce the contraction rate of neonatal mouse ventricular myocytes through Gα_i_ activation (Figure 3A). Furthermore, methoctramine prevents the reduction in the contraction rate of neonatal mouse ventricular myocytes evoked by TCDCA, TDCA, and GDCA [57]. Moreover, TDCA attenuates the contraction in aortic rings induced by phenylephrine, which is a selective α1-adrenergic receptor agonist. This TDCA-induced reduction in aortic ring contraction is reduced in aortic rings from mAChR M3 knockout mice, indicating that TCDCA-induced relaxation is mediated by mAChR M3 [58]. Furthermore, DCA suppresses the expression of cancer stem cell makers, such as aldehyde dehydrogenase 1, cluster of differentiation 166 (CD166), and Myc, through mAChR M3 in HCoEpiC, normal human colonic epithelial cells [59,60].

### 3.4. MRGPRX4

MRGPRX4, a member of the Mas-related, G-protein-coupled receptor family, is primarily expressed in sensory nerves and is related to nociception and itch. The human Mas-related, G-protein-coupled receptor family consists of eight members, including MRGPRX 1, 2, 3, and 4, and MRGPR D, E, F, and G. The mouse ortholog of MRGPRX4 is MrgprA1 [61]. MRGPRX4 and MrgprA1, receptors for BAs and bilirubin, are predominantly expressed in the primary sensory neurons of the dorsal root ganglia [62,63,64,65,66]. Primary sensory neurons of the dorsal root ganglia are associated with itch and transmit itch as well as pain from the skin to the spinal cord [67]. MRGPRX4 is coupled to Gα_q_, and its activation leads to the production of diacylglycerol and IP_3_, which causes Ca^2+^ release (Figure 3B). DCA, TDCA, UDCA, CDCA, TCDA, and CA activate MRGPRX4, leading to Ca^2+^ release [63,64]. Furthermore, DCA, UDCA, and CA activate DRG neurons expressing MRGPRX4 [63,64]. Mice expressing MRGPRX4 scratched significantly following the injection of DCA, TDCA, UDCA, or CDCA into the nape [64]. Furthermore, intradermal injection of DCA, CDCA, TCDCA, and CA results in a robust itching sensation in healthy subjects [63]. It has been shown that 3-sulfated DCA (DCA-3S) also activates MRGPRX4 more potently than DCA. Sulfation at the 3-position also enhances the agonistic activity of LCA, TLCA, CA, GUDCA, or TUDCA toward MRGPRX4 [62].

A recent study reported that MRGPRX4 interacts with receptor activity-modifying proteins (RAMPs) [68], which include RAMP1, RAMP2, and RAMP3 [69]. RAMPs were first identified as single-pass transmembrane proteins that associate with receptors of the calcitonin peptide family, which comprises calcitonin (CT), calcitonin gene-related peptide, adrenomedullin, amylin, and procalcitonin. The CT receptor (CTR) and CTR-like receptor (CLR) are receptors for calcitonin family members. The specificity for CT family members is determined by the combination RAMPs and CTR or CLR [69,70,71,72,73]. Currently, more than 40 GPCRs have been reported to interact with RAMPs [68]. MRGPRX4 can associate with RAMP2 and RAMP3. Coexpression of MRGPRX4 and RAMP2 or RAMP3 reduces inositol phosphate-1 (IP1) accumulation induced by DCA, TDCA, and UDCA. RAMP2 is a more potent inhibitor than RAMP3. Although RAMP2 causes a slight decrease in total MRGPRX4 expression levels, RAMP2 causes a strong decrease in the cell surface expression of MRGPRX4 (Figure 3B). In contrast, RAMP3 had no effect on both the total and cell surface expression of MRGPRX4 [68]. Prediction of the MRGPRX4–RAMP2 complex structure revealed that the extracellular domain of RAMP2 appears to “cap” the extracellular portion of MRGPRX4 [65]. Therefore, it is considered that RAMP2 changes the intracellular localization of MRGPRX4 and inhibits its expression on the cell membrane surface, thereby restricting the function of MRGPRX4 as a cell membrane surface receptor. However, the mode of inhibition of MRGPRX4 by RAMP3 is unknown, and the detailed mechanism of inhibition of MRGPRX4 by RAMPs needs to be elucidated. In addition, the possibility that RAMPs affect GPCRs other than MRGPRX4, which are ligands of BAs, needs to be examined.

### 3.5. Bitter Taste Receptors

Many mammals, including humans, detect sweet, umami, bitter, sour, and salty tastes using taste receptors. By combining these five basic tastes, we can recognize the taste of food and determine its nutritional content, toxicity, or spoilage. Sweet, umami, and bitter tastes are detected by different GPCRs in taste receptor cells [74]. In humans, three GPCR subtypes involved in sweet and umami taste perception have been identified: taste receptor type 1 member 1 (T1R1), member 2 (T1R2), and member 3 (T1R3), which together constitute the taste receptor type 1 (T1R) family [75]. The sweet taste receptor functions as a heterodimer of T1R2 and T1R3, whereas the umami taste receptor is formed by a heterodimer of T1R1 and T1R3 [74,76]. In contrast, bitter taste is recognized by a diverse family of receptors known as taste receptor type 2 (TAS2R) family. Sour and salty tastes are detected by ionotropic receptors [77]. Recent studies have shown that BAs can bind to and activate bitter taste receptors [78,79,80]. Currently, 26 TAS2Rs have been identified in humans and 35 in mice [81,82]. In addition, BitterDB holds approximately 700 compounds that have been reported to activate human TAS2Rs [81]. Furthermore, TAS2R is expressed in various tissues other than the oral cavity, including the gastrointestinal tract [83,84].

**Figure 4:**
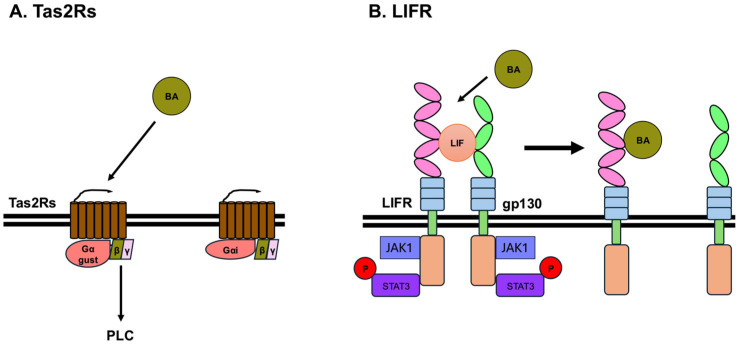
Taste receptor type 2 (TAS2Rs) and LIFR with gp130. (**A**) The activation of TAS2R leads to the dissociation of Gα-gustducin from βγ-subunit. The dissociated βγ-subunit activates phospholipase C (PLC), leading to the release of Ca^2+^. CA, TCA, GCA, LCA, TLCA, DCA, CDCA, and UDCA activate five human bitter receptors, namely TAS2R1, TAS2R4, TAS2R14, TAS2R39, and TAS2R46, and five mouse bitter receptors, namely mTas2R105, mTas2R108, mTas2R123, mTas2R126, and mTas2R144. (**B**) LIF interacts with LIFR at D3 and D4 and with gp130 at D2 and D3. DCA, TDCA, GDCA, 3-oxoDCA, LCA, T LCA, GLCA, and 3-oxoLCA act as antagonists against the binding of LIFR with LIF. These bile acids inhibit the association between LIF and LIFR by binding to the D3 region of LIFR.

HEK293T-Ga16gust44 cells, which are HEK293T cells that stably express the chimeric G-protein Gα16gust44 [85], were transiently transfected with human, mouse, chicken, or frog bitter taste receptors and these bitter taste receptors responded to several BAs [78,79]. CA, TCA, GCA, LCA, TLCA, DCA, CDCA, and UDCA activate five human bitter receptors, which are TAS2R1, TAS2R4, TAS2R14, TAS2R39, and TAS2R46, and five mouse bitter receptors, which are mTas2R105, mTas2R108, mTas2R123, mTas2R126, and mTas2R144 [78,79]. Mouse Tas2R105, Tas2R108, Tas2R123, Tas2R126, and Tas2R144 are structural orthologs of human TAS2R10, TAS2R4, TAS2R14, TAS2R41, and TAS2R140, respectively. Human TAS2R1, TAS2R39, and TAS2R46, are structural orthologs of mouse Tas2R119, Tas2R139, and Tas2R136/120, respectively [83,86]. TCA and GCA activate all five human bitter receptors, whereas CA and TLCA activate four, DCA, CDCA, and UDCA activate two receptors, and LCA activates only one receptor. LCA and TLCA are the most potent BAs that activate human bitter taste receptors [79] (Figure 4A). TAS2R1 is activated by all eight of these BAs and is therefore the least selective receptor among these five human bitter taste receptors. TAS2R4, TAS2R14, TAS2R39, and TAS2R46 react with 6, 5, 3, and 3 BAs, respectively, out of the 8 BAs mentioned above. The binding site of BAs in TAS2R14 has been analyzed, and the putative binding site of BAs in TAS2R14 is the orthosteric binding site of TAS2R14 [78,80]. TAS2R14 couples to Gα-gustducin and Gα_i_, and the most well-known signaling pathway of TAS2R is IP_3_/Ca^2+^ signaling pathway. Upon TAS2R activation, Gα-gustducin dissociates from the βγ subunit. The dissociated βγ-subunit activates phospholipase Cβ2, which activates the IP_3_/Ca^2+^ signaling pathway [87] (Figure 4A). Many types of bitter taste receptors, including TAS2R14, are expressed in tissues of the nervous, endocrine, respiratory, and immune systems [83,84], suggesting that BAs may affect human health and disease via bitter taste receptors. Currently, it has been reported that bitter taste receptors function as BA receptors at the cellular level, and further progress is expected to uncover the physiological and pathological functions of BAs and bitter taste receptors.

### 3.6. Leukemia Inhibitory Factor Receptor (LIFR)

Leukemia inhibitory factor (LIF) is one of cytokines and a member of interleukin 6 family [88]. It has been recently reported that several BAs, such as DCA, TDCA, GDCA, 3-oxoDCA, LCA, TLCA, GLCA, and 3-oxoLCA, affect LIFR [89]. LIFR heterodimerizes with glycoprotein 130 (gp130), also known as CD130, IL-6 receptor subunit β, or IL-6 signal transducer. Although the heterodimeric receptor complex of LIFR and gp130 do not have kinase activity, the cytoplasmic domains of the receptor complex of LIFR and gp130 bind to Janus kinase 1 (JAK1), JAK2, and tyrosine kinase 2. The main kinase activated by LIF is considered to be JAK1 [90]. Binding of LIF to the receptor complex of LIFR and gp130 activates JAK1, leading to the phosphorylation of STAT3. Phosphorylated STAT3 forms a dimer and enters the nucleus, where it acts as a transcription factor and regulates the expression of its target genes [91]. In addition, JAK1 activated by LIFR activates the PI3K/AKT and MAPK signaling pathways [92]. The extracellular domains of LIFR include an N-terminal, cytokine-binding homology region (CHR1, D1, and D2), immunoglobulin (Ig)-like domain (D3), the second CHR2 (D4 and D5), and three membrane-proximal fibronectin type III domains (D4 to D6). In contrast, gp130 includes an N-terminal Ig-like domain (D1), a CHR (D2 and D3), and three membrane-proximal FNIII domains (D4 to D6). Cryo-EM structures of the complex of LIFR, gp130, and LIF reveal that LIF interacts with LIFR at D3 and D4 and with gp130 at D2 and D3 [93,94] (Figure 4B).

DCA, TDCA, GDCA, 3-oxoDCA, LCA, TLCA, GLCA, and 3-oxoLCA act as antagonists, inhibiting the binding of LIFR with LIF [89]. LIF-induced STAT3 phosphorylation is inhibited by these eight BAs. The IC50 values of TDCA, GDCA, 3-oxoDCA, and 3-oxoLCA were 1.6–2.5 μM, which is similar to the concentrations that can activate TGR5 [19,89]. These BAs inhibit the association between LIF and LIFR by binding to loop 2 (303-NPGRVTALVGPRAT-316), and to loop 3 (332-KRAEAPTNES-341) in the D3 region of LIFR (Figure 4B). Furthermore, LCA derivatives, TLCA, GLCA, and 3-oxoLCA, prevent the growth effects of LIF in MKN45 (human gastric cancer cell line), MIA PaCa-2 (human pancreas cancer cell line), HepG2 (human liver cancer cell line) and Caco-2 (human colon cancer cell line). DCA and its derivatives, including TDCA, GDCA, and 3-oxoDCA, inhibit the growth effects of LIF in Caco-2 and MKN45 cells [89]. Furthermore, LIF influences the growth and development of various cancers, including breast, gastric, pancreatic, kidney, prostate, and ovarian cancers [92,95,96]. Therefore, LCA and DCA derivatives function as LIFR antagonists, which may therefore lead to the development of therapeutic agents that inhibit LIF-induced tumor growth and progression.

## 4. Potential Medicines Targeting Cell Surface Receptors for Bile Acids

In recent years, targeting BA receptors has emerged as a promising strategy for treating various diseases, including those affecting the nervous, endocrine, immune systems, as well as cancer. Therefore, the development of specific BA receptor ligands has significant therapeutic potential. Currently, several small molecules are being investigated as potential BA receptor ligands for their efficacy in the treatment of various diseases.

Several natural compounds with potency comparable to LCA have been identified as effective TGR5 ligands. These include other BAs [97] and triterpenoids, such as oleanolic acid and ursolic acid [98,99,100]. Furthermore, a number of synthetic compounds with potency comparable to LCA have also been reported. INT-777 [38,101] and BAR501 [102] are semisynthetic BA derivatives and have been widely studied. Various other synthetic ligands for TGR5 have also been reported [103,104,105,106,107,108,109]. However, systemic activation of TGR5 agonists has been associated with adverse side effects, particularly the inhibition of gallbladder emptying, which may promote gallstone formation [110,111,112]. Therefore, it is necessary to develop drugs that do not induce the side effects of systemic TGR5 agonists and to establish methods for delivering drugs to target tissues. Recent studies have demonstrated that OM8 [105] and compound 19 [108] do not affect gallbladder volume and bile weight. suggesting their potential to mitigate the gallbladder-related side effects evoked by TGR5.

JTE-013 is an antagonist that binds to the same site on S1PR2 as TβMCA [49]. In contrast, CYM-5520 is a noncompetitive allosteric agonist of S1PR2 [49,113]. Because CYM-5520 binds to a different site, it can bind with TβMCA to S1PR2 and modulate TβMCA activity. Thus, these compounds can affect the various physiological functions mediated by S1PR2 activation.

Methoctramine, a mAChR M2 antagonist, prevents the inhibition of the contraction of myocytes induced by TCA, TCDCA, TDCA, and GDCA [56,57].

Fospropofol, fosphenytoin, dexamethasone phosphate, and MS47134 are identified as selective agonists for MRGPRX4, and these molecules elicit itching in mice [114,115]. Cryo-EM structural analysis of fospropofol-bound MRGPRX4 with Gq revealed that crucial amino acids (W158, Y250, and Y254) form a hydrophobic pocket in MRGPRX4, while L83 is key for agonist recognition [114]. These results contribute to understanding the mechanism of BA-induced pruritus and support the development of targeted therapies. Furthermore, EP547 is identified as an antagonist for MRGPRX4, and clinical trials of EP547 for the treatment of cholestatic pruritus are currently underway [116,117].

Flufenamic acid, papaverine, glycyrrhizic acid, diphenhydramine, and rubusoside act as agonists for TAS2R14. Binding region of these five ligands to TAS2R14 is the same pocket in the transmembrane domain 3 and 6 of TAS2R14 [118]. Tas2Rs, including TAS2R14, are expressed in various tissues and can elicit diverse physiological and pathophysiological responses [83,84]. Therefore, elucidating the binding modes and mechanisms of action of ligands, including BAs, at TAS2R will lead to the discovery and development of novel drugs.

## 5. Conclusions

Various metabolites of BAs are synthesized by intestinal bacteria. In addition to glycine- and taurine-conjugated BAs, bacteria also produce BAs conjugated with other amino acids [119,120,121], resulting in a diverse repertoire of BAs present in the body [120,122]. The mechanism of action of BAs as signaling molecules has been elucidated by many researchers, and there are many nuclear and cell membrane receptors that have been identified as receptors for BAs. TGR5, SIP2R, and mAChRs have long been known as cell membrane receptors for BAs. Recent structural analysis of TGR5 by Cryo-EM has revealed the switching mechanism of the Gs and β-arrestin signaling system in TGR5, offering insights that may aid in the development of TGR5-targeted therapies. Moreover, MRGPX4, bitter taste receptors, and LIFR have been reported as novel BA receptors. Because there are many different types of bitter receptors, it is plausible that they respond to the metabolic products of several BAs and to BAs conjugated with various amino acids. Furthermore, MRGPRX4 interacts with RAMPs, which modulate its response to BAs. Therefore, it is possible that BA-specific GPCRs may also be affected by RAMPs, and this creates more complex effects of BAs. Thus, different receptors correspond to different BAs, underscoring their complex physiological actions. Continued research on BAs and their receptors is likely to deepen our understanding of various disease mechanisms and facilitate the development of novel drugs and therapies for those diseases.
